# Dr. Hall's Enquiries into the Nervous System

**Published:** 1840-04

**Authors:** Marshall Hall

**Affiliations:** London


### THE RESULTS OF DR. MARSHALL HALL'S ENQUIRIES INTO THE NERVOUS SYSTEM, AS STATED BY HIMSELF.

We have readily acquiesced in Dr. Hall's request that we should insert a concise statement of his present views and claims. We cannot accuse ourselves, however, of having either neglected them or treated them unfairly. We have followed him, step by step, in his researches,—not giving an immediate assent to his opinions when they seemed to us deficient in solid foundation,—but not condemning them as absurd or untenable. We have pointed out instances in which Dr. Hall was anticipated by previous experimenters; but we have not accused him of making an unfair use of their researches. And, in ultimately giving our assent to his peculiar doctrines, because additional evidence has been adduced in their support, we have expressed, in the most decided manner, our conviction of their importance. If our readers will turn to pp. 101-4 of the present volume, they will see the doctrine of the *distinct spinal system* and its peculiar functions, enunciated by Dr. Hall in his *first*, *second*, *fourth*, and *fifth* propositions, set forth as fully as the object of that article permitted. We there speak of the discovery of these as constituting the *third great era* in the history of neurology, and as almost entirely the work of Dr. M. Hall. We said "almost," because the *anatomical* evidence in its support, which we deem of much importance, was *not* furnished by him, but by others. At a subsequent part of the same article (pp. 115-6), we speak of the discovery of *this distinct system* as even more the work of Dr. M. Hall than the discovery of the distinctness of the motor and sensitive nerves was of Sir C. Bell. We cannot see what more Dr. Hall can require from us. We should have thought, too, that he would have regarded *our* accordance with his doctrines as more valuable than that of individuals or journals whose assent was given hastily, and without close examination of their merits. But we fear that he is so convinced that we always *must be opposing* him, that he does not know when we coincide with him.

In regard to Dr. Hall's *third* proposition, we must confess ourselves at present unable to see its importance. We know *nothing* at present of the mode in which any impressions—sensory or volitional, incident or motor—are conveyed by the nerves. To refer them all to one common head is all that we can do; for anything we know, the modes of action may be the same in all these instances, or it may be different. The terms *vis nervosa, nervous action*, and the like, can only be used, therefore, in the same vague and general sense as *vitality*, or any other abstract character of that nature.

Some of Dr. Hall's later researches (of which the results have been only just published) we have noticed in our present Number (p. 438); and to the remainder we shall return when they are incorporated in the Third Memoir, which we understand Dr. Hall has in preparation, and which we shall take the first opportunity of noticing. Those of our readers who feel an interest in this question may advantageously refer to a *former* statement of his claims by Dr. M. Hall, to which we gave admission (Vol. III., p. 577). It appears to us that Dr. H. now relinquishes (as having been previously set forth by others) what he then claimed as his own.

*To the Editor of the British and Foreign Medical Review.*

Sir,—Will you do me the favour to insert the following paragraph relative to what I myself consider to be the results of my own enquiries into the Nervous System?

It is well known,—

1. That a series of *experimental* phenomena were observed in decapitated animals by Redi, Whytt, Legallois, Blane, &c.

2. That similar phenomena and certain *sympathetic actions* were referred, by Prochaska, Dr. Alison, and Mr. Mayo, to certain nerves termed sentient, certain parts of the brain and spinal marrow, and motor nerves.

3. That Blane distinctly stated that the motions observed in a decapitated animal did not depend upon sensation.

It is obvious that, whatever my own claims may be, therefore, they cannot include any of these points. What, then, are they?

I claim, as the peculiar result of my own investigations, the discovery,

1. That in the same theca vertebralis there is, beside the *cord* of cerebral, tactile, and voluntary nerves, a distinct organ and nervous centre, which I have designated the true spinal *marrow*.

2. That in the same neurilemma with the tactile and voluntary nerves, and also exclusively of these, there is a *system* of incident and reflex nerves, of which the true spinal marrow is the centre, the combiner, &c.

3. That the *principle of action* in this subdivision of the nervous system is the *vis nervosa* of Haller, acting in directions and according to laws unknown and even denied before.

4. That through this same subdivision of the nervous system, and on this same principle of action, *all ingestion* and *all egestion* in the animal economy are effected—and as consequences: 1, The preservation of the individual; and 2, the continuance of the species.

5. That there is indeed a special system of *respiratory* nerves; but that this includes incident and excitor nerves, as well as the respiratory nerves of Sir Charles Bell, and that it is only a part of the more general excito-motory system.

6. That, in a word, every act of ingestion and of egestion is a *spinal* act, resulting from *excito-motory* influences of the vis nervosa, urged in *reflex* directions, along the incident and reflex *nerves*, to which I have adverted.

7. That upon the same anatomy, motor principle, &c., the whole class of spasmodic diseases, their pathology and their therapeutics, also depend. I may add to these statements, as the results of more recent enquiries, that I believe it to be established:

1. That, whilst *volition* acts upon the cerebrum and through cerebral nerves alone, *emotion* acts upon the medulla oblongata, and the excito-motory nerves.

2. That the *irritability* of the muscular fibre depends upon the true spinal marrow and the ganglia, exclusively of the cerebrum.

3. That as consequences of these laws, in hemiplegia the *opposite* side of the body is at once *paralyzed* to *volition*, but *agitated* by *emotion;* that the irritability is augmented in *cerebral*, but diminished in *spinal* paralysis.

If I should be wrong in asserting these claims, and any critic candidly point out my error, I shall be at once obliged and ready to confess my mistake. But it is useless to continue to disprove claims which I never made, whilst my real claims are allowed to pass unnoticed.

May I take this opportunity of recording a fact, observed during the late autumn, and communicated at that time to some of my friends? If we take the *libellula*, or dragon-fly, remove the head, separate the thorax, and divide the abdomen into three parts, we have *five* distinct portions of the insect manifesting rhythmic respirations. In each we must consequently have the "analogue" of the medulla oblongata in the vertebrated class. I propose to pursue the enquiry when the spring again brings out these interesting insects. It remains to be determined whether each ganglion singly and all unitedly be analogous to the medulla oblongata, quoad respiration, associated with their incident and reflex excito-motory nerves, and to trace the nervous system of deglutition, defecation, &c.

I am, Sir, your obedient servant,

*London; Feb.* 21, 1840. MARSHALL HALL.

